# Fate of carotenoid-producing *Bacillus aquimaris* SH6 colour spores in shrimp gut and their dose-dependent probiotic activities

**DOI:** 10.1371/journal.pone.0209341

**Published:** 2018-12-21

**Authors:** Huong Thi Nguyen, Tham Thi Nguyen, Huong Thi Thu Pham, Que Thi Ngoc Nguyen, My Thi Tran, Anh Hoa Nguyen, Tuan Nghia Phan, Ha Thi Viet Bui, Hien Thi Thanh Dao, Anh Thi Van Nguyen

**Affiliations:** 1 Key Laboratory of Enzyme and Protein Technology, VNU University of Science, Vietnam National University, Hanoi, Thanh Xuan, Hanoi, Vietnam; 2 ANABIO Research & Development JSC, Van Khe urban, Ha Dong, Hanoi, Vietnam; 3 Traditional Pharmacy Department, Hanoi Pharmacy University, Hanoi, Vietnam; Loyola University Chicago, UNITED STATES

## Abstract

*Bacillus aquimaris* SH6 spores produce carotenoids that are beneficial to white-leg shrimp (*Litopenaeus vannamei*) health. However, the optimal dose and mechanisms behind these effects are not well understood. We investigated the fate of SH6 spores in the gut of *L*. *vannamei*. Shrimp were divided into six groups administrated with either feed only (negative control) or SH6 spores at 5 × 10^6^ CFU/g pellet (high dose, SH6 spore-H group), 1 × 10^6^ CFU/g pellet (medium dose, SH6 spore-M group), 2 × 10^5^ CFU/g pellet (low dose, SH6 spore-L group), astaxanthin at 0.5 mg/g pellet (Carophyll group), or carotenoids from SH6 vegetative cells at 5 μg/g pellet (SH6 carotenoid group). The growth rate was highest in SH6 spore-H (3.38%/day), followed by SH6 spore-M (2.84%/day) and SH6 spore-L (2.25%/day), which was significantly higher than the control (1.45%/day), Carophyll (1.53%/day) or SH6 carotenoid (1.57%/day) groups. The astaxanthin levels (1.9–2.0 μg/g shrimp) and red-colour scores (21–22) in SH6 spore-H/M were higher than the control (astaxanthin: 1.2 μg/g shrimp; red score: 20) or SH6 spore-L, but lower than the Carophyll and SH6 carotenoids. Feeding with medium and high doses of SH6 spores after 28 days resulted in respective 1.3-2-fold increases in phenol oxidase activity and 8–9 fold increases in *Rho* mRNA expression compared to the control and low dose group. The live-counts of SH6 in the gut gradually increased during the 28-day feeding period with SH6 spores at different concentrations, starting from 4.1, 8.2, and 5.4 × 10^4^ CFU/g gut at day 1 and reaching 5.3, 5.1, and 4.4 × 10^5^ CFU/g gut in the SH6-H/M/L groups, respectively, at day 28. Gut microbiota became more diversified, resulting in a 2-8-fold increase in total bacterial live-counts compared to the controls. SH6 spore germination was detected by measuring the mRNA expression of a specific sequence coding for SH6 amylase at 4 h, reaching saturation at 24 h. Our results confirm that SH6 spores colonize and germinate in the gut to improve the microbial diversity and boost the immune system of shrimp, exhibiting beneficial effects at >1 × 10^6^ CFU/g pellet.

## Introduction

For sustainable shrimp aquaculture, the most useful and safe method consists of adjusting the diet of shrimp by feeding with probiotic-supplemented food [1–4]. Probiotic supplements can improve the production yield in shrimp aquaculture by adjusting and strengthening the microbiota in the gut. Moreover, they also promote bio-competition by creating adverse conditions for disease causing microbes through the modulation of gut microbiota, as well as adjusting the immune response of the host [5–9]. In terms of boosting the innate immunology of shrimp, the following four factors have been commonly analysed: Ras-like GTP binding protein (*Rho*), *Ran* binding protein (*Ran*), phenol oxidase (PO) and superoxide dismutase (SOD). The first and second factors, *Rho* and *Ran*, belong to the GTPase protein group and are known to be involved in shrimp phagocytosis [10], where *Rho* is involved in superoxide formation and opsonisation, two indispensable mechanisms of phagocytosis in shrimp [11,12]. Therefore, *Rho* and *Ran* expression is a marker for the ability of cells to defend against pathogen invasion. The third and fourth factors, PO and SOD activity, represent the level of immune response in shrimp. SOD activity plays a vital role in protecting cells from oxidative stress due to its ability to catalyse cellular reactions and maintain the lowest possible levels of ROIs (reactive oxygen immediate) in cells. SOD activity is proportionally related to increased immune response in shrimp [13]. Conversion of pro-phenol oxidase (Pro-PO) to active form phenol oxidase (PO) is highly correlated with the formation of melanin as well as the innate immune system [14].

In recent years, the commercial export value of white-leg shrimp (*Litopenaeus vannamei*) has started to depend not only on weight but also on the shrimp’s astaxanthin levels and red-colour score. Astaxanthin levels in shrimp are considered an important indicator of quality, including redness and survival rate [15]. Therefore, the improvement of the astaxanthin levels and the redness of white-leg shrimp is of great interest for scientists and producers in the field of shrimp aquaculture [16–18]. As a result, many studies and reports have focused on screening carotenoid-producing bacteria spores originated from soil, sea-water and human faeces. For example, *B*. *marisflavi* and *B*. *aquimaris* strains produce red pigments [18], whereas some *B*. *firmus* strains produce pink pigments. A variable yellow-orange pigmentation has been found in a number of species including *B*. *indicus*, *B*. *cibi*, *B*. *vedderi*, *B*. *jeotgali* and *B*. *pseudofirmus* [16,19]. Among these studies, there is only one recent publication by our group on the screening of a *Bacillus sp*. strain producing carotenoids isolated from shrimp gastrointestinal tracts [20]. In that study, the best carotenoid producing orange-pigmented *B*. *aquimaris* SH6 strain was isolated and screened from the gut of white-leg shrimp. The strain was characterized for its potential probiotic effect on shrimp health, as indicated by increased PO activity, weight gain and improved red colour score and astaxanthin levels [20]. Nevertheless, there are still other factors regarding the application and mechanism of probiotic activity that need to be addressed, including the identification of the optimal feeding regime of SH6 spores as cost-effective feed supplements, the verification of whether the SH6 orange spores can colonize and germinate into vegetative cells during the transition of feed through the shrimp intestine in order to produce carotenoids and whether the persistence of SH6 effects in the gut microbiota confer beneficial effect. To answer these questions, we analysed the probiotic activity of SH6 in a dose-dependent manner using the main indicators of health and yield of the shrimp host. These included the growth rate and survival rate of shrimp, the red score and astaxanthin levels in the shrimp body, four immune-associated indicators of PO and SOD activity and the expression of *Rho* and *Ran* mRNA. To understand the mechanism of probiotic activity, we also investigated the colonization and germination of *B*. *aquimaris* SH6 spores within the epithelium gut in a time- and dose-dependent manner.

## Materials and methods

### Preparation of pigmented *B*. *aquimaris* SH6 spores and carotenoid extract from *B*. *aquimaris* SH6 vegetative cells

An orange-pigmented *B*. *aquimaris* SH6 strain isolated from the gut of white-leg shrimp and characterized from a previous study was used for growing spores [20]. In brief, SH6 spores were formed in Difco Sporulation Medium (DSM), pH 8, at 33°C and shaking for 48 h in a flask. Spore counts were determined by total cell forming unit after heat-count for 20 min at 60°C to remove residual vegetative cells. The methodology was slightly modified from the method described by *Feavers et al*.[21]. SH6 carotenoid was extracted from SH6 bacterial cells grown in Tryptone Soy Broth (TSB) at 33°C for 24 h. The total cells were collected and pulped in nitrogen liquid, before adding cod liver oil and sonicating using Labsonic M ultrasonic homogenizer (Sartorious, Goettingen, Germany) [19]. The upper phase was collected for carotenoid level determination by measuring at absorbance 480 nm (A_480_) following the method described by Ngo *et al*. [20]. The spores and the carotenoid extract from SH6 bacterial cells (hereafter named as “SH6 carotenoids”) were then stocked, aliquoted, and stored at -20°C for further analysis.

### Preparation of supplemented feed for trials in white-leg shrimp

Commercial shrimp feed pellet (Universal President, Tainan, Taiwan) was first autoclaved to kill any live probiotic strains and was then used as the basal diet for supplementing with either SH6 spores at three different concentrations, Carophyll Pink (powder containing 10% synthesized astaxanthin, DSM, Heerlen, the Netherlands) or SH6 carotenoids; a similar method has been reported elsewhere [20]. For the three experimental groups, the feed was mixed with SH6 spores at the following final concentrations: 5 × 10^6^ CFU/g pellet (high concentration, labelled as SH6 spore-H group), 1 × 10^6^ CFU/g pellet (medium concentration, labelled as SH6 spore-M), 2 × 10^5^ CFU/g pellet (low concentration, labelled as SH6 spore-L). For the two positive control groups, the feed was mixed with either Carophyll at a final concentration of 0.5 mg/g pellet or SH6 carotenoids extracted from SH6 vegetative cells at a final concentration of 5 μg/g pellet. Finally, 100 g of the supplemented feed pellets, either for the experimental or positive groups, were coated with 10 ml cod liver oil Seven Seas (Merck Consumer Health, Darmstadt, Germany). As a negative control, the feed pellets were coated with only cod liver oil. All the prepared feed pellets for the six experimental groups were then aliquoted and stored at -20°C for use in the shrimp trials.

### Oral feeding of white-leg shrimp

Twenty-five-day-old white-leg shrimp (*L*. *vannamei*) was procured from the Qui Kim Aquaculture Research Center, Duong Kinh District, Hai Phong City, Vietnam, weighing approximately one gram were used for probiotic treatment on a laboratory scale. The approval of an animal care and use protocol for conducting trials on shrimp was not required since they are invertebrates. Trials in white-leg shrimp followed biosecurity guidelines of the Department of Aquaculture, Vietnam Ministry of Agriculture and Rural Development.

To test the probiotic activity of SH6 spores, such as weight gain, astaxanthin levels and body colour, the immunostimulatory effects as well as the colonization of SH6 in the shrimp gut were evaluated and blind trials were conducted. Shrimp were divided into six groups (*n* = 70) and each group was housed in two separate glass 45-litre cubic-shaped tanks (*n* = 35/tank) containing 25 litres of artificial sea water and equipped with an air supply system. The shrimp were maintained under the following conditions: 26–28°C, pH 7.5–8.5, D.O. ≥4 mg/L, 16 ppt salinity. For oral administration, six types of feed prepared were used, as described above (negative control, Carophyll, SH6 spore-H, SH6 spore-M, SH6 spore-L, SH6 carotenoids). The shrimp were fed with 2–3 g feed pellets per day continuously for 28 d.

To test the germination of SH6 spores in the shrimp gut, the shrimp were housed in glass 45-litre cubic-shaped tanks (*n* = 30/tank) and cultured under the same conditions as above. For oral administration, feed supplemented with SH6 spores at a high dose of 1 × 10^8^ CFU/g pellet were used. The shrimp were fed with only a single dose of 2–3 g feed pellets and cultured for a further 7 d.

### Measurement of weight gain, body colour score and astaxanthin levels in shrimp

For the determination of weight gain in the shrimp, twenty shrimp (*n* = 20) from each group at day 28 were taken to measure the average weight and then compared to that of day 0 using the following equation: $\text{GR}(\text{growth}\;\text{rate})=\frac{final\;weight\;-initial\;weight}{days\;of\;feeding}\;\text{x}\;100\%$. The survival rate (%) of the shrimp was calculated using the following formula: SR = 100 × (n_t_/n_o_), where SR is the survival rate, n_t_ is the number of shrimp at the time t and n_o_ is the initial number of shrimp.

For measurement of the body colour score, five shrimp (*n* = 5) from each group were taken after 28 d and boiled to observe the level of red colour using the Roche index, using SalmoFan standard colour as a reference [20]. For measuring the astaxanthin levels in shrimp muscle (μg/g shrimp), five shrimp (*n* = 5) from each group were also taken at day 28. Astaxanthin was extracted and identified following the method described by Ngo *et al* [20].

### Measurement of *Rho* and *Ran* mRNA expression and PO and SOD activity

To measure the *Rho* and *Ran* mRNA expression and the PO and SOD activity, three samples (3 shrimp per sample) from each group were taken at the following time points: 0 and 28 d. For the measurement of *Rho* and *Ran* mRNA, total RNA was extracted from 10 mg of shrimp muscle using a RNeasy Mini Kit (Qiagen, Stockach, Germany) followed by cDNA synthesis using a M-MLV Reverse Transcriptase Kit (Enzynomics, Incheon, Korea) according to the manufacturer’s instructions. Specific primer sets for *Rho* and *Ran* genes were designed and described in Table 1. Real-time PCR using SYBR Green assay kit (TOPreal qPCR 2X PreMIX, Enzynomics, Korea) was performed to amplify the 180-bp specific sequence for *Rho* cDNA with a single melting peak at 80°C ± 1°C. As this assay was non-specific for *Ran* amplification, real-time PCR using Taqman FAM-probe (see Table 1) and engineered high-speed hot-start Taq polymerase (RbTaq Fast qPCR 2X PreMIX, Enzynomics, Korea) was used to amplify the 280-bp specific sequence for *Ran* cDNA. As an internal control, *β-actin* primers were used to amplify a 122-bp specific sequence [22]. The primer and probe sequences are shown in Table 1. Finally, the relative gene expression of *Rho* and *Ran* mRNA was measured based on the 2^-ΔΔCt^ value, in which C_t_ was the threshold cycle, following the method of Livak *et al*. [23]. PO activity was measured by spectrophotometry to record the dopachrome transformation from L-dihydroxyphenylalanine (L-DOPA) at A_490_, using the method described by Hermander-Lopez *et al*. and Ngo *et al*. [20,24]. PO activity was presented by the change of A_490_ value over 60 min.

**Table 1:** Specific primers for real-time RT-qPCR.

No	Target gene	cDNA (bases)	Primer and probe	Reference
1	*Rho*	180	Fw: 5’—gaaaatattccagaaaaatggacg—3’ Rv: 5’—tttatcttctcagccatgttacga—3’	This study
2	*Ran*	280	Fw: 5’—aggtccatcctctcgttttc—3’ Rv: 5’—gcccatctacgagggatat—3’ P: /5HEX/atactgctg/ZEN/gccaagagaagttgggagg/3IABkFQ/	This study
3	*β-actin*	122	Fw: 5’—gcccatctacgagggatat—3’ Rv: 5’—ggtggtcgtgaaggtgtag—3’	[22]
4	*BaqA*-SH6	110	Fw: 5’—gggtaccatggctattggattgagga—3’ Rv: 5’—actttcatatcccgtttatgtgcttct—3’ P: /56/FAM/accgaagaa/ZEN/catttcggctccatgaagac/3IABkFQ/	This study

### Live-counting of *B*. *aquimaris* SH6 and total bacteria in the intestinal track of shrimp

To perform live-counts of SH6 and determine the total bacterial population in the shrimp gut at different time points (0, 1, 3, 7, 14 and 28 d), shrimp (*n* = 3 per time points) were sacrificed at these times and their guts were vigorously suspended in 0.9% NaCl. Samples were serial diluted with 0.9% NaCl before being plated on LB agar, TSA or MRS agar (Oxoid, Hampshire, UK). The plates were incubated at 33°C for 24 to 48 h depending on the medium used to obtain individual colonies with typical morphology and colour. To identify the bacterial colonies at the species level, colonies with different morphology were sub-cultured in LB or TSB medium to isolate total genomic DNA. Using the extracted DNA, colonies were identified based on 16S rRNA sequencing and BLAST analysis following the method described by Ngo *et al*. [20]. Three colonies with the same morphology and colour were used to repeat the species identification three times. Then, the number of colonies with the same morphology and colour grown on the plates were taken from each group and were counted.

### Design of degenerate and specific primers for amplification of *BaqA*-SH6 gene

**Fig 1:**
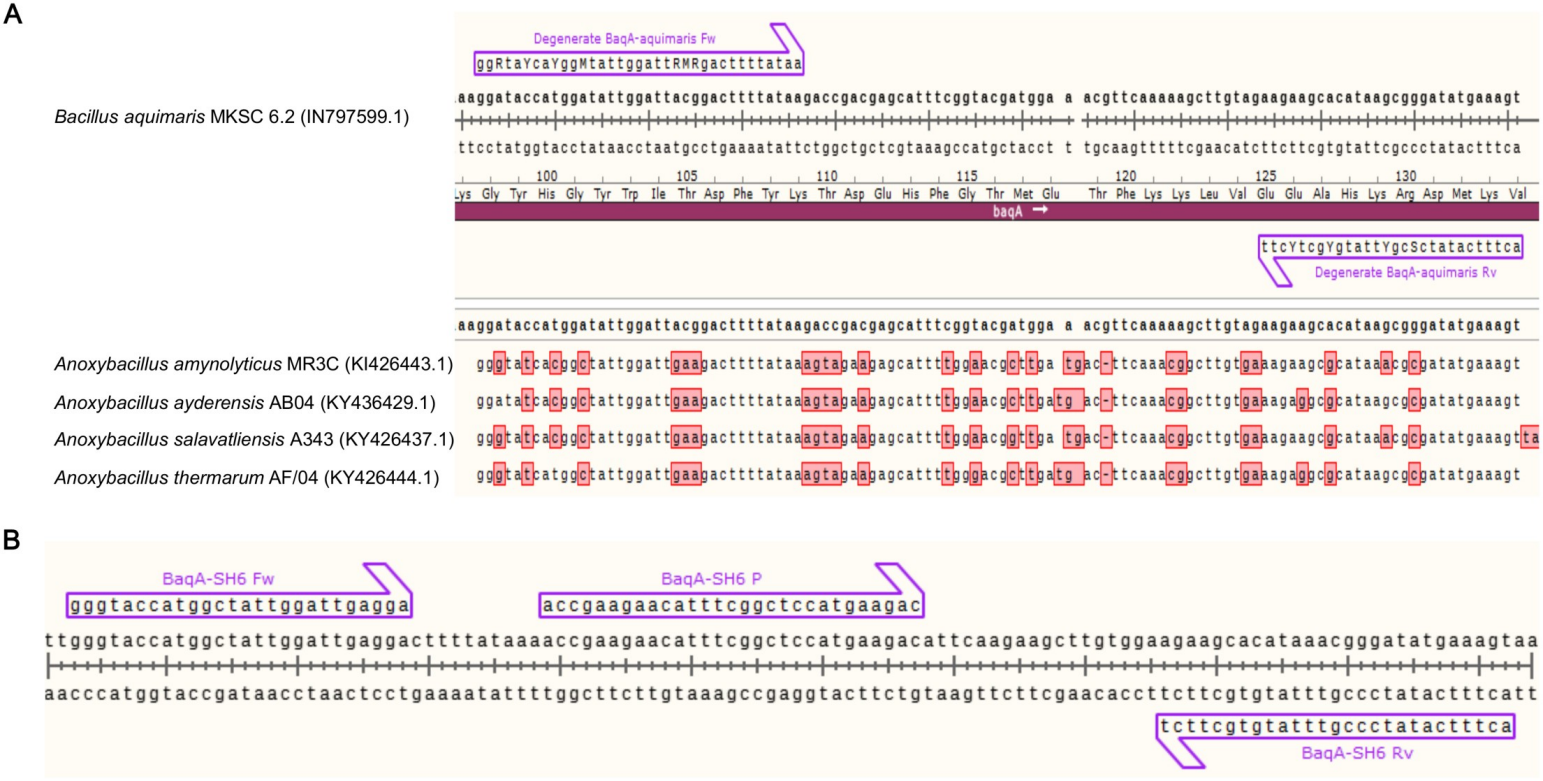
Primer design for specific amplification of *BaqA*-SH6 α-amylase gene. (A) Alignment between a 110 bp DNA sequence specific for the α-amylase coding gene of *B*. *aquimaris* MKSC 6.2 and four sequences of strains belonging to *Anoxybacillus* genus (Acession numbers were placed next to the strain names), featured with two degenerate primers and coding amino-acid sequence. (B) 110 bp DNA sequence of *BaqA*-SH6 gene coding for α-amylase of the *B*. *aquimaris* SH6 strain, featured with specific primers and probes for use in real time RT-qPCR.

*BaqA*-SH6 degenerate Fw and Rv primers were designed based on the only available DNA sequence coding for *BaqA*, the gene coding for α-amylase of *B*. *aquimaris* MKSC 6.2 (Accession No. JN797599.1), according to Puspasari *et al*. [25], in alignment with several genes coding for α-amylase of *Anoxybacillus* sp. strains, which have been reported to share similarity with *B*. *aquimaris* and belong to a new subfamily of glycosyl hydrolase family GH13 [26] (Fig 1). The degenerate primers were placed at the most homologous sequence of the *BaqA* genes (Fig 1A), allowing a 110 bp DNA sequence of both *B*. *aquimaris* SH6 and another *Bacillus* pigmented *B*. *aquimaris* SH1 strain (Accession No. KF443805) [20] to be amplified with PCR for 1 cycle of 95°C for 10 min, 35 cycles of denaturation at 95°C for 30 s, annealing at 60°C for 30 s and elongation at 72°C for 30 s. To determine the specific primers for *BaqA*-SH6, the PCR product from the SH6 strain was ligated into p-TOP TA V2 cloning vector (Enzynomics, Incheon, Korea), and the target sequence was identified by DNA sequencing using either M13 Fw or M13 Rv primers. Finally, specific primers and a probe to amplify the 110 bp sequence of *BaqA*-SH6 were designed (Fig 1B) and are shown in Table 1. Specific amplification of the 110 bp sequence of *BaqA*-SH6 was tested under the above PCR condition using the designed specific primers and DNA templates extracted from different sources: (i) vegetative cells of *B*. *aquimaris* SH6; (ii) vegetative cells of two reference strains *B*. *subtilis* PY79, *B*. *indicus* HU36, and of two *Bacillus* pigmented strains *B*. *aquimaris* SH1 and *B*. *marisflavi* SH8 (Accession No. KF443806) of intestinal origin [20]; (iii) shrimp gut from SH6-spore M/L groups and negative control group.

### Measurement of *BaqA*-SH6 mRNA expression in shrimp gut

The shrimp (*n* = 3 per time point) were sacrificed at 0 h, 3 h, 4 h, 6 h, 12 h, 1 d, 2 d, 4 d and 7 d after the start of feeding to collect the gut and then extract total RNA using a RNeasy Mini Kit (Qiagen, Stockach, Germany). Real-time RT-qPCR was used to measure the expression of *BaqA*-SH6, which is an indicator of the level of *B*. *aquimaris* SH6 germination, and was performed using the extracted RNA as templates, specific primers and the TaqMan probe following the method published by Cartman *et al*. [27], with modifications in the design of specific primers and probes for the gene coding α-amylase of the SH6 strain. The one-step master mix used for real-time RT-qPCR was TOPreal One-step RT qPCR Kit—RT430 (Enzynomics, Korea) at the following PCR conditions of: 1 cycle of 50°C for 30 min and initial denaturation at 95°C for 10 min, 45 cycles of denaturation at 95°C for 10 s, annealing and elongation at 60°C for 30 s. Detection of fluorescence signal was turn on since cycle 10. For data analysis, the relative mRNA expressions of *BaqA*-SH6 at different time points were calculated based on the comparative C_ta_ (threshold cycle) values [28]. Germination rates (%) were calculated using the following equation: 2^-(Cta-Ct20)^ x 20% where C_ta_ was the C_t_ at certain time point; C_t20_ was the C_t_ of RNA samples extracted from one fifth (20%) of SH6 vegetative cells (100% germination in LB medium) with the same live-count as SH6 detected in shrimp gut at day 2.

### Statistical analysis

The data for astaxanthin levels, weight gain, bacteria counts, *Rho* mRNA expression levels, and PO activity among the six treatment groups were compared using a Student’s t-test at significance levels of 0.05 and 0.01. Statistical analyses were performed using Analysis ToolPak in Microsoft Excel. An F-test for two-sample variance was used before performing a t-test for two unpaired samples. ANOVA single factor analysis was used for comparison of more than two samples.

## Results

### Weight gain in white-leg shrimp was improved by supplementing with SH6 spores in a dose-dependent manner

**Fig 2:**
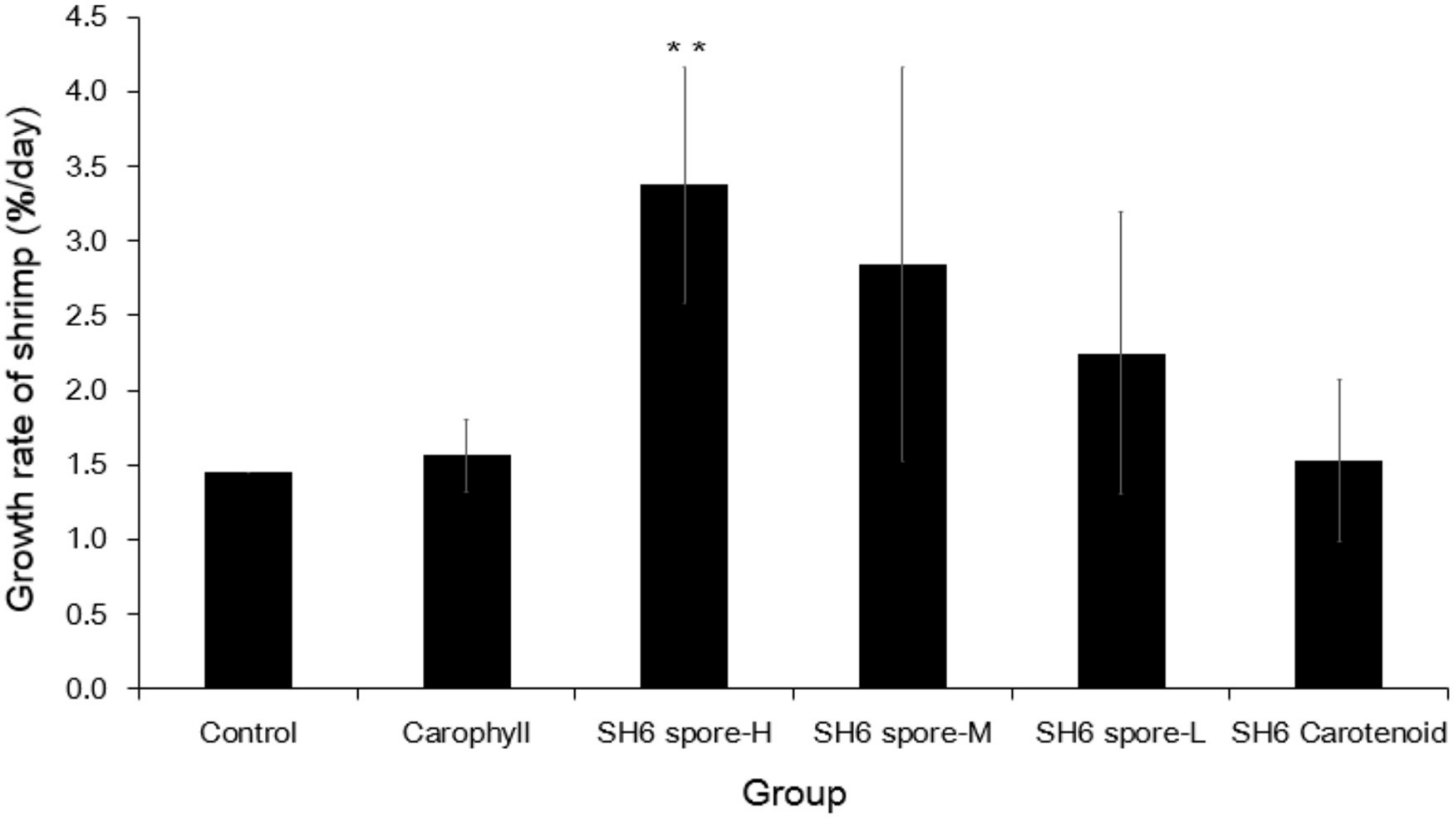
Growth rate of *L*. *vannamei* after 28 d feeding with SH6 spores. Experiment includes negative control, Carophyll (astaxanthin: 0.5 mg/g pellet), SH6 carotenoid (SH6 carotenoids: 5 μg/g pellet), and SH6 spore-H/M/L groups (SH6 spores at 5 × 10^6^, 1 × 10^6^, 2 × 10^5^ CFU/g pellet, respectively). Growth rate factor is indicated by weight gain (%) per day. Data are presented as arithmetic means and error bars are standard deviations (*n* = 20). *P* values were generated using Student’s t-test for multiple comparisons to the control. P values were generated using Student’s t-test for comparison to the control (**P <0.01).

The first experiment was conducted to determine the optimal dose of SH6 spore supplement for improving weight gain in the shrimp. Our data revealed that after 28 d feeding, there was an apparent disparity between the spore and non-spore groups, and between the spore groups at different doses. The shrimp in the three groups, SH6 spore-H, SH6 spore-M, and SH6 spore-L, all gained weight faster than the control, the Carophyll and the SH6 Carotenoid group, where *P* <0.01. Based on the weight at day 0 and day 28, we calculated the growth rate (GR) of shrimp administrated with SH6 spores and other groups as described in the Methods. The growth rate was the highest in the SH6 spore-H group (3.38%/day) (*P* <0.01), followed by the SH6 spore-M group (2.84%/day) and the SH6 spore-L group (2.25%/day). The Carophyll and SH6 carotenoid groups showed less marked differences (1.53–1.57%/day) compared to the control group (1.45%/day) (Fig 2). This result suggests that SH6 spores improved shrimp weight gain in a dose-dependent manner. We also determined whether SH6 spores supported the survival of shrimp in all six groups. However, since the shrimp culture conditions at the laboratory scale were of a high enough quality that the survival rates in all the groups were similar or greater than 90% after 28 days of feeding, it was not possible to evaluate this effect.

### Astaxanthin levels and pigmentation in white-leg shrimp were improved by supplementing with SH6 spores at high and medium doses

**Fig 3:**
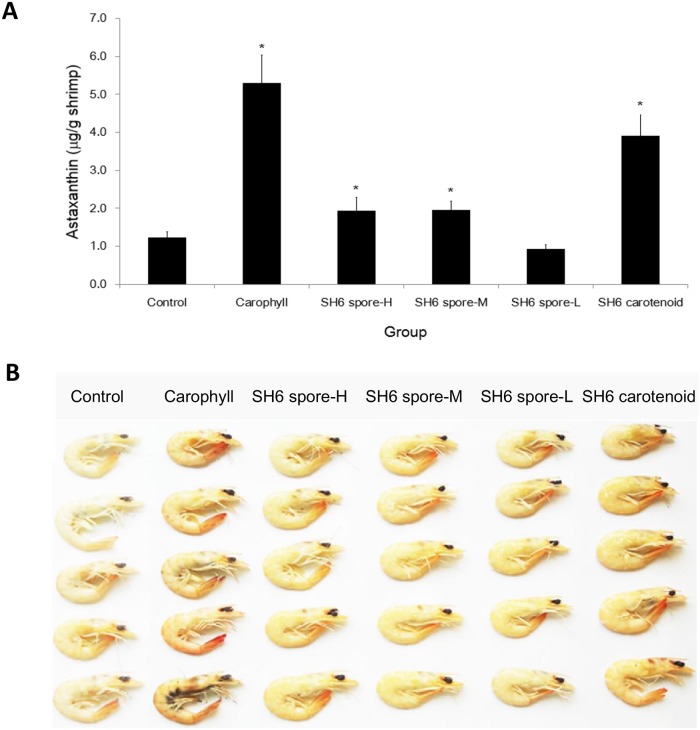
Astaxanthin concentration and colour in *L*. *vannamei* after 28 d feeding with SH6 spores. Experiment includes negative control, Carophyll (astaxanthin: 0.5 mg/g pellet), SH6 carotenoid (SH6 carotenoids: 5 μg/g pellet), and SH6 spore-H/M/L groups (SH6 spores at 5 × 10^6^, 1 × 10^6^, 2 × 10^5^ CFU/g pellet, respectively). (A) Astaxanthin concentrations in shrimp muscles (μg/g shrimp). Data are presented as arithmetic means and error bars are standard deviations (*n* = 5). *P* values were generated using Student’s t-test for multiple comparisons to the control (*P <0.05; **P <0.01). (B) Image of boiled shrimps and their variable colour scores indicating the levels of red pigmentation (*n* = 5).

We further investigated whether or not carotenoids produced by the *B*. *aquimaris* SH6 strain could be converted to astaxanthin in the shrimp in a dose-dependent manner. In other words, the optimal dose of SH6 spore supplement for improving astaxanthin levels and body colour in shrimp was determined. After carrying out feeding at three different doses, as described in the Materials and methods, we extracted astaxanthin from the muscle of the shrimp and measured the astaxanthin levels of shrimp from each group. As shown in Fig 3A, the concentration of astaxanthin (μg/g shrimp) in the sample of the Carophyll and SH6 carotenoid groups was clearly higher (5.3 and 3.9 μg/g shrimp, respectively) than that of the control group (1.2 μg/g shrimp), where *P* <0.05. The astaxanthin levels in the SH6 spore-H (1.9 μg/g shrimp, *P* <0.05) and SH6 spore-M (2.0 μg/g shrimp, *P* <0.05) groups were significantly higher than those of the control group, however not comparable to the Carophyll and SH6 carotenoid groups. On the other hand, the low dose of SH6 spores was not effective in improving astaxanthin levels in the shrimp, as indicated by 0.9 μg/g shrimp in the SH6 spore-L group.

To identify the relationship between the presence of astaxanthin in shrimp tissue and shrimp colour, we evaluated the effects of SH6 probiotics on the pigmentation of white-leg shrimp in a dose-dependent manner. After 28 d of feeding, the colour of the shrimp after boiling was compared among the six groups (Fig 3B). As predicted, the Carophyll and SH6 carotenoid groups had the highest red-colour score (score: 22–23), followed by the SH6 spore-H/M groups (score: 21–22), the SH6 spore-L group (score: 20–21) and the negative control group (score: 20). In conclusion, our data shows that SH6 spores at high and medium doses, but not at the low dose, could significantly improve astaxanthin levels and slightly improved the body colour of the shrimp, compared to the control.

### Increased immune-related *Rho* mRNA expression levels and phenol oxidase activity in shrimp by supplementing with SH6 spores at high and medium doses

**Fig 4:**
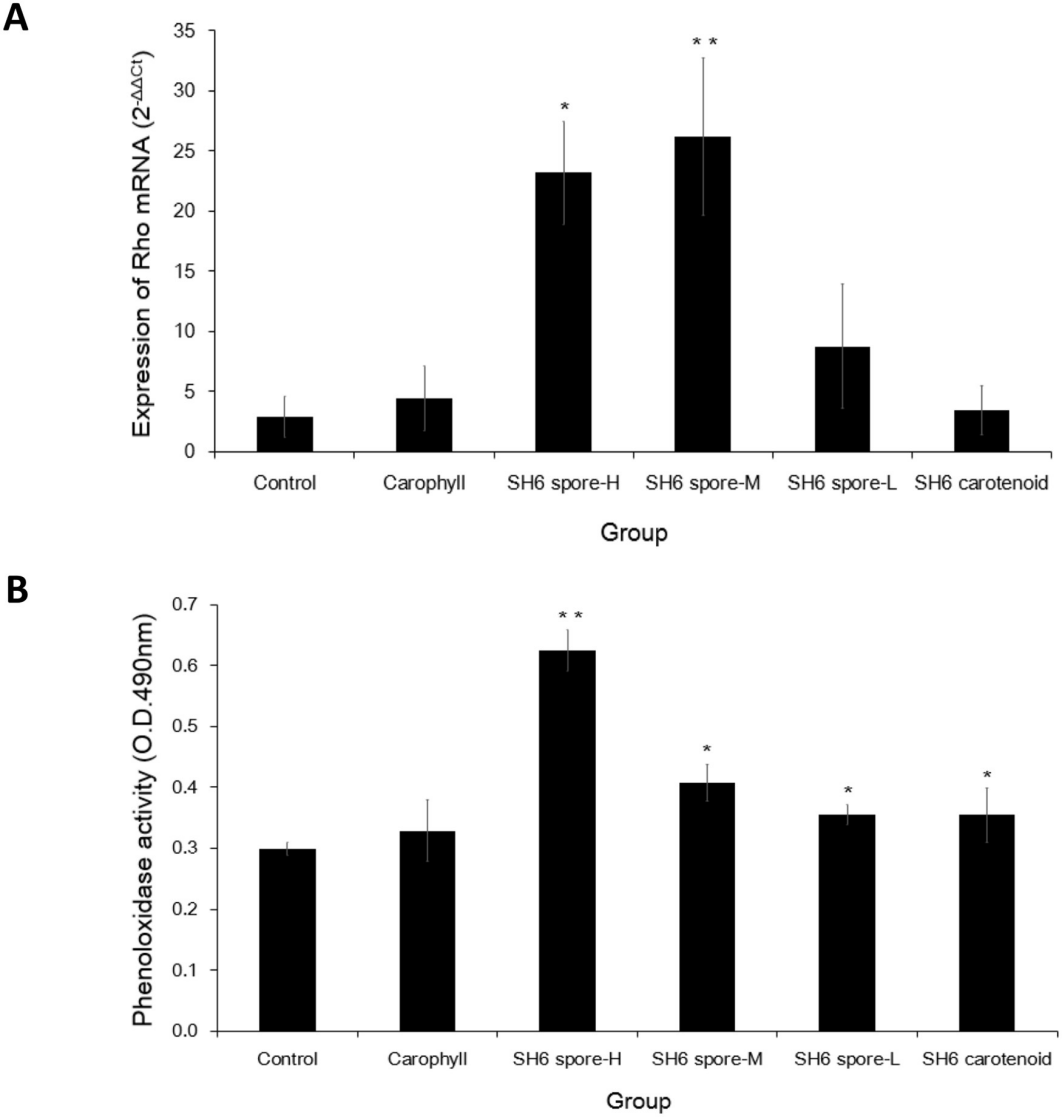
*Rho* mRNA expression and Phenoloxidase activity of *L*. *vannamei* after 28 d feeding with SH6 spores. Experiment includes negative control, Carophyll (astaxanthin: 0.5 mg/g pellet), SH6 carotenoid (SH6 carotenoids: 5 μg/g pellet), and SH6 spore-H/M/L groups (SH6 spores at 5 × 10^6^, 1 × 10^6^, 2 × 10^5^ CFU/g pellet, respectively). (A) Gene expression level of *Rho* (Ras-like protein) gene is indicated by 2^-ΔΔCt^ value. (B) Phenoloxidase (PO) activity is indicated by OD_490nm_. Data are presented as arithmetic means and error bars are standard deviations (*n* = 3). *P* values were generated using Student’s t-test for multiple comparisons to the control (*P <0.05; **P <0.01).

To determine whether SH6 spores could induce factors reflecting the innate immune response in shrimp in a dose-dependent manner, we analysed the first common immune-stimulation molecular marker in shrimp, the levels of mRNA expression of *Rho* gene, among the six shrimp groups (*n* = 3 per group). *Rho* mRNA expression at day 28 was measured by real-time PCR using SYBR Green specific for the *Rho* gene (see Methods). The data are presented in Fig 4A and show that there was a significantly increased *Rho* mRNA expression in the SH6 spore-H (2^-ΔΔct^ = 23.2, 8.1-fold, *P* <0.05) and SH6 spore-M (2^-ΔΔct^ = 26.2, 9.2-fold, *P* <0.01) groups compared to the negative control group (2^-ΔΔct^ = 2.8). The expression levels in the SH6 spore-L (2^-ΔΔct^ = 8.8, 3.0-fold), positive Carophyll (2^-ΔΔct^ = 4.4, 1.5-fold) and SH6 carotenoid (2^-ΔΔct^ = 3.4, 1.2-fold) groups were also higher than the negative control group; however, the differences were not statistically significant. The second immune-stimulation molecular marker measured was *Ran* mRNA expression. However, we found that *Ran* mRNA was over-expressed only in the two positive control groups fed with Carophyll (2^-ΔΔct^ = 367) and SH6 carotenoids (2^-ΔΔct^ = 1459), which were about 6-fold and 25-fold higher than that of the negative control (2^-ΔΔct^ = 58), respectively. This factor was expressed at a normal level in the three SH6 spore H/M/L groups (2^-ΔΔct^ values were about 38–54) (S1 Fig).

In addition to the two genetic markers, we also tested the changes in PO and SOD activity, since these are commonly considered enzymatic markers and are associated with the immune response in shrimp. As shown in Fig 4B, the PO activity of the shrimp in the SH6 spore-H group was improved by over 2-fold after a 28-day continuous feeding period, compared to the negative control group (A_490_: 0.63 vs. 0.30 respectively, *P* <0.01). Even though the PO values among the SH6 spore-M (1.33 fold), SH6 spore-L (1.16 fold) and SH6 carotenoid groups were only slightly higher than the negative control (1.16 fold), the differences were statistically significant (*P* <0.05). In terms of SOD activity, we unexpectedly did not observe any significant changes in SOD activity during the 28-day feeding period in all six groups. Taking all the data together, we demonstrated that SH6 spores at high and medium doses could induce an increase in the two molecular markers *Rho* mRNA and PO activity, which reflect the shrimp innate immune system, while the spores at the low dose did not significantly affect either of these two markers. By contrast, feeding the shrimp directly with synthesized astaxanthin or extracted SH6 carotenoids significantly induced *Ran* mRNA over-expression, but did not effectively induce the remaining two factors (PO, and SOD).

### Increased persistence of *B*. *aquimaris SH6* spores in the shrimp gut and improvement of bacteria microbiota in shrimp supplemented with SH6 spores

**Fig 5:**
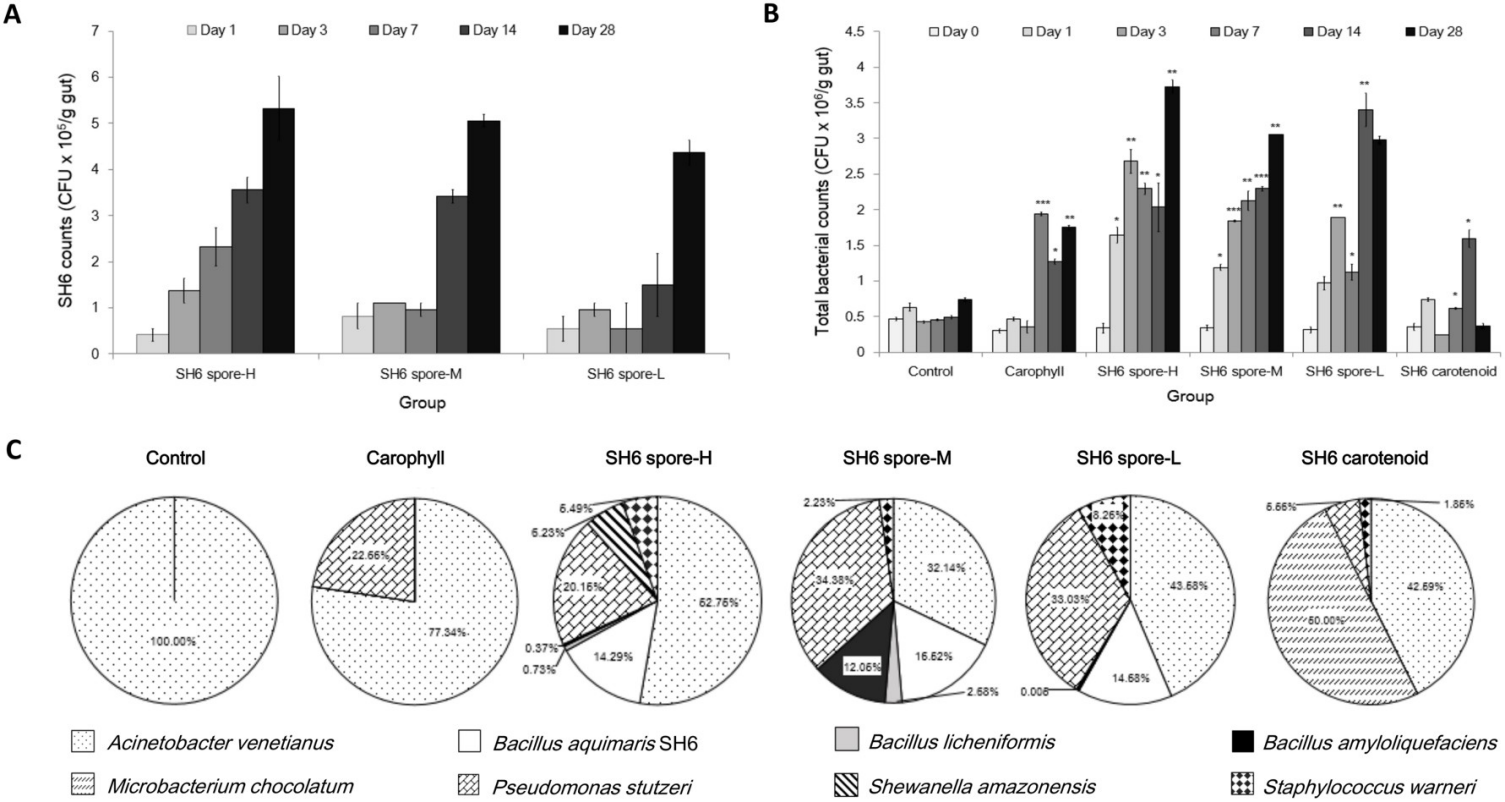
Live counts of *B*. *aquimaris* SH6 and total bacteria in the gut of *L*. *vannamei* during 28 d feeding with SH6 spores. Experiment includes negative control, Carophyll (astaxanthin: 0.5 mg/g pellet), SH6 carotenoid (SH6 carotenoids: 5 μg/g pellet), and SH6 spore-H/M/L groups (SH6 spores at 5 × 10^6^, 1 × 10^6^, 2 × 10^5^ CFU/g pellet, respectively). (A) Time-course live counts of SH6 in shrimp gut at day 0, 7, 14, 28 among the three SH6-spore H/M/L groups. (B) Time-course live counts of total bacteria in shrimp gut at day 0, 1, 3, 7, 14 and 28 among the six experimental groups. (C) Percentage of live counts of major bacterial species detected in shrimp gut at day 28 among the six experimental groups.

To determine the mechanism of probiotic activity in SH6 spores, we first investigated whether the spores could adhere to the epithelium intestine of shrimp and whether this had an effect on the gut microbiota. Thus, the live counts of *B*. *aquimaris* SH6 and other bacterial strains recovered in the shrimp intestines were determined by plating samples of excised GI-tracts onto selective agar (see Methods). Our data revealed that the number of SH6 colonies increased over time in all three SH6 spore-H/M/L groups from day 1 (4.1 × 10^4^, 8.2 × 10^4^, 5.4 × 10^4^ CFU/g gut, respectively) to day 28 with respective values of 5.3, 5.1 and 4.4 × 10^5^ CFU/g gut for the SH6 spore-H/M/L groups. Even though the final output live-counts at day 28 of the three SH6 spore feeding groups were not very different, the rate of increased live counts of SH6 in the shrimp gut, especially at days 3 and 7, was quickest in the SH6 spore-H group, followed by that of the SH6 spore-M and SH6 spore-L groups (Fig 5A). To determine whether the increase in SH6 colonies in the shrimp gut is effective in improving the live counts and diversity of the gut bacteria, we identified the colonies at the species level and counted other non-pigmented bacteria colonies in the shrimp gut of the SH6 spore H/M/L groups during feeding time. We found that the total bacterial live counts of these SH6 spore feeding groups (within 3.0–3.7 × 10^6^ CFU/g gut) were also increased over time, and were found to be significantly higher than those found in the negative control, Carophyll, or SH6 carotenoid groups (within 0.4–1.7 × 10^6^ CFU/g gut) (*P* <0.01, *P* <0.001, *P* <0.05, respectively) (Fig 5B). Consistently with the speed of SH6 increase, the rate of total bacterial counts increase was also highest in the SH6 spore-H group at days 3 and 7, followed by the SH6 spore-M and SH6 spore-L group. The data of 16S rRNA sequencing for species identification revealed that the SH6 spore-containing groups exhibited more diversified useful bacterial populations than the negative, Carophyll or SH6 carotenoid groups. As shown in Fig 5C, there were seven major bacterial strains closely related (ID ≥99%) to the species *Acinetobacter venetianus*, *Microbacterium chocolatum*, *Pseudomonas stutzeri*, *B*. *licheniformis*, *B*. *amyloliquefaciens*, *Shewanella amazonensis*, and *Staphylococcus warneri* identified in the shrimp gut of the SH6 spore-H group, and six and five major useful species in the shrimp gut of the SH6 spore-M and L groups, respectively. By contrast, there were only four (*A*. *venetianus*, *M*. *chocolatum*, *P*. *stutzeri*, and *S*. *warneri*), two (*A*. *venetianus* and *P*. *stutzeri*) and one (*A*. *venetianus*) major bacterial species found in the shrimp gut of the SH6 carotenoid, Carophyll and the negative control groups, respectively. In conclusion, our data indicate that the persistence of SH6 spores in the shrimp gut increased over the duration of the oral administration of SH6 spores and proportionally to the feeding dose. We also concluded that the increased SH6 live-counts were consistent with an improvement in the total live counts and diversity of the gut bacteria, in a time- and dose-dependent manner.

### Germination of *B*. *aquimaris* SH6 spores adhered on shrimp gut

**Fig 6:**
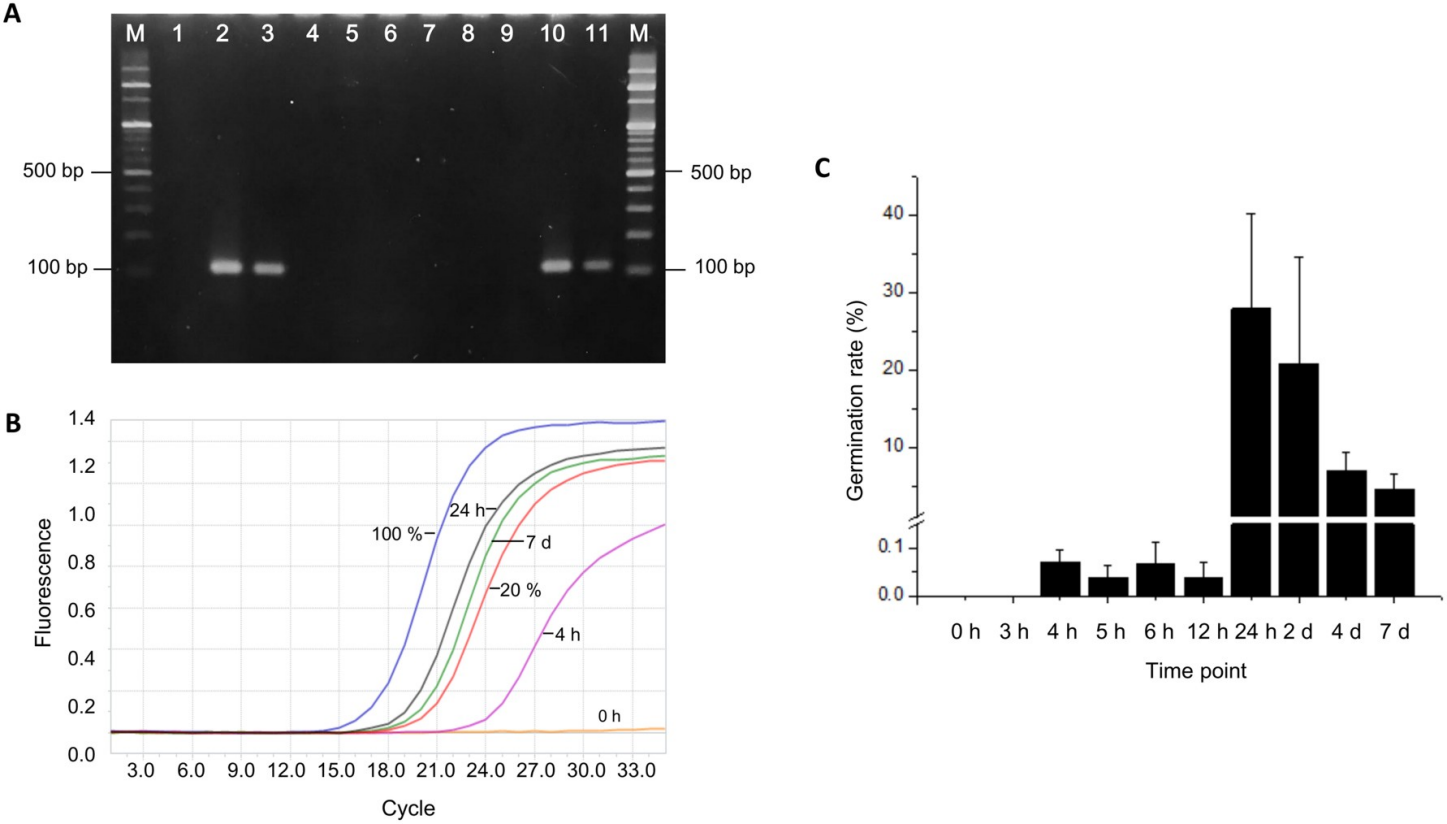
Expression of *BaqA*-SH6 mRNA in the gut of *L*. *vannamei* during 7 d feeding with SH6 spores. (A) Agarose-gel electrophoresis of 110 bp PCR product specific for *BaqA*-SH6 gene using specific primers and DNA templates extracted from different samples. Lane 1: water only-negative control; lane 2, 3: bacterial cells of *B*. *aquimaris* SH6; lane 4: *B*. *subtilis* PY79; Lane 5: *B*. *indicus* HU36; Lane 6: *B*. *aquimaris* SH1 (Accession No. KF443805); lane 7: *B*. *marisflavi* SH8 (Accession No. KF443806); lane 8, 9: shrimp gut from negative control; lane 10, 11: shrimp gut from SH6-spore M/L groups; M: 100 bp DNA ladder. (B) Shrimp was fed with SH6 spores at 1 × 10^8^ CFU/g pellet. RNA was extracted from shrimp gut at 0h, 3 h, 4 h, 5 h, 6 h, 12 h, 24 h, 2 d, 4 d, 7 d after spore administration, and then was used as templates for real-time RT-qPCR measuring mRNA expression of the 110 bp sequence of *BaqA*-SH6. Representative amplification curves using RNA extracted at time points: 0, 4, 5, 6, 12, 24 h, 2 d, 4 d and 7 h in comparison to positive controls of 20% germination. (C) Germination levels (%) of SH6 spores in shrimp gut at different time points after spore administration.

From the above experiment, it is evident that SH6 spores are able to reside in the epithelium intestine of shrimp. Next, we investigated whether the SH6 spores were able to germinate into vegetative cells to produce digestive enzymes, such as amylase, during the few hours of transition of feed through the shrimp intestine. We measured the mRNA expression levels of *BaqA*-SH6 (the gene coding for α-amylase of *B*. *aquimaris* SH6) in the shrimp gut after feeding with SH6 spores at a single dose of 1 × 10^8^ CFU/g pellet by real-time RT-qPCR using designed specific primers and the FAM-labelled TaqMan probe (see Methods). Firstly, we confirmed the specificity of the primers designed with the aim to amplify gene coding for amylase of only *BaqA*-SH6, and not of other bacteria in the shrimp gut. The result of DNA electrophoresis of PCR bands shown in the Fig 6A indicated that specific 110 bp PCR bands were amplified only if using template DNA extracted from the two samples: SH6 bacterial cells (Fig 6A, lane 2, 3; repeated twice) and the shrimp fed with SH6 spore-M/L groups (Fig 6A, lane 10, 11; repeated twice). By contrast, there was no band detected when template DNA extracted from the reference strains, such as *B*. *subtilis* PY79 (Fig 6A, lane 4), *B*. *indicus* HU36 (Fig 6A, lane 5), *B*. *aquimaris* SH1 (Fig 6A, lane 6), *B*. *marisflavi* SH8 (Fig 6A, lane 7) and shrimp gut from the negative control group (Fig 6A, lane 8, 9) were used.

**Fig 7:**
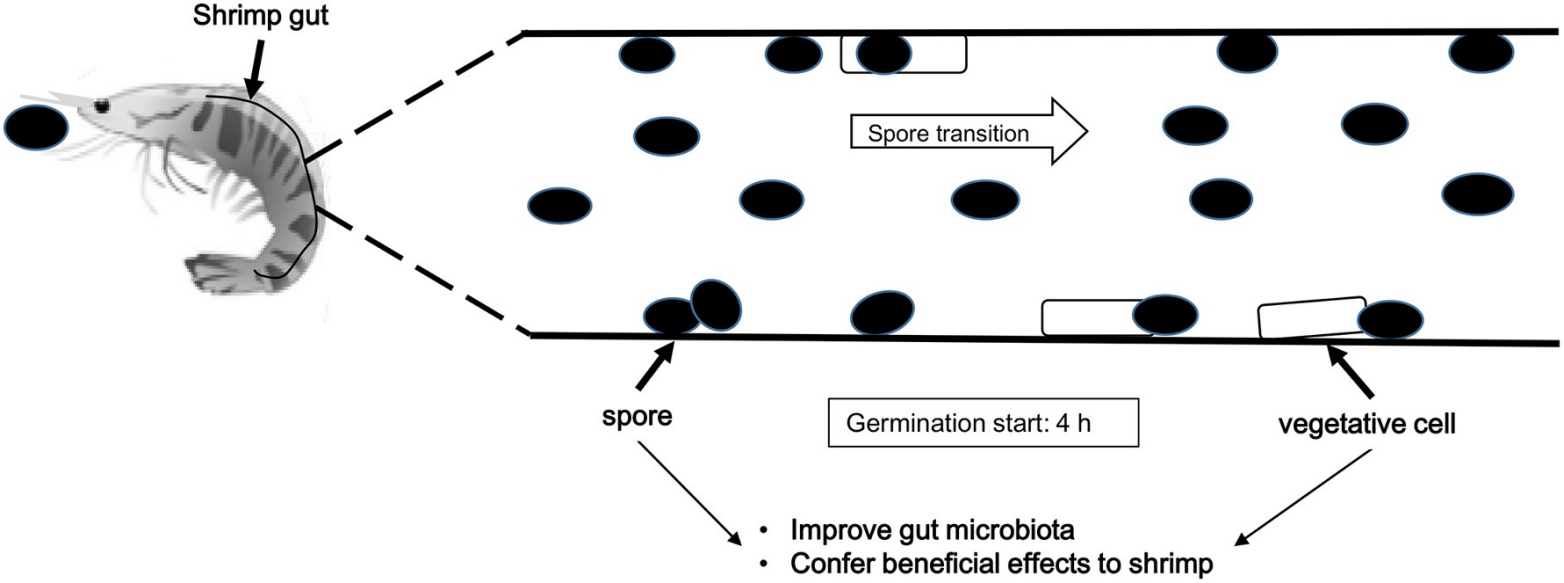
Model for fate of SH6 spores in shrimp gut. After feeding SH6 spores to white-leg shrimp, majority of the spores transit quickly through the shrimp gut. However, minority of spores adhere and accumulate onto the intestinal epithelium, then germinate into vegetative cells after 4 h feeding. The colonized spores and vegetative cells of SH6 strain play a key role in improving gut microbiota and conferring multiple beneficial effects to the host.

Using specific primers, we designed a FAM-labelled TaqMan probe specifically for *BaqA*-SH6 (Table 1) and carried out measurements of *BaqA*-SH6 mRNA expression by RT-qPCR. Representative amplification curves at different time points (0 h, 3 h, 4 h, 5 h, 6 h, 12 h, 24 h, 2 d, 4 d and 7 d) are displayed in Fig 6B, showing that the C_t_ values of the amplification curves decreased, that is mRNA expression levels increased, over time. To qualitatively measure the percentages of spore germination at different time points, we compared the mRNA expression levels of samples to the mRNA extracted from either 20% or total SH6 vegetative cells (100% germination in LB medium) with the same live-count as SH6 detected in shrimp gut at day 2. Interestingly, we found that mRNA expression started at 4 h and remained at very low levels (0.03–0.07%) until 12 h. The highest germination level (27.87%) was observed only at 24 h (day 2), but had a tendency to decrease at days 4 and 7 (Fig 6C). As the transition of spores through shrimp gut lasts less than four hours, this data showed that the expression of *BaqA*-SH6 α-amylase gene, which is an indicator of SH6 spore germination, was due to the contribution of SH6 spores which colonized the intestinal epithelium but not the spores transiting freely through the shrimp gut. In addition, when there was lack of nutrition supply, the expression level of mRNA decreased at days 4 and 7. Based on this data, we propose a model for fate of SH6 spores in shrimp gut, as presented in Fig 7.

## Discussion

In this study, we fed shrimp different SH6-spore doses in order to determine an optimal dose for the cost-effective use of feed supplements for application in shrimp aquaculture. Determining an optimal dose is particularly important for pigmented *Bacilli* spores because the production of pigmented *Bacilli* spores such as *B*. *aquimaris* SH6 or *B*. *indicus* HU36 results in a lower yield and is therefore more costly than the production of non-pigmented spores (*B*. *subtilis* or *B*. *licheniformis*). We chose three representative doses: 2 × 10^5^ CFU/g pellet (low), 1 × 10^6^ CFU/g pellet (medium) and 5 × 10^6^ CFU/g pellet (high), which fall within the range of 1 × 10^5^ to 1 × 10^7^ CFU/g pellet, which are the recommended doses for probiotics in shrimp aquaculture [29]. As a matter of fact, we obtained a significantly increased growth rate in white-leg shrimp in a dose-dependent manner of SH6 spore feeding, especially at high and medium doses. On the other hand, SH6 spores supplied at a low dose, and the groups of Carophyll and SH6 carotenoids could not improve growth rate in white-leg shrimp significantly (Fig 2). This suggests that the higher the dose of SH6 spores, the greater the weight gain obtained.

Astaxanthin is a carotenoid found in shrimp which results in the red body colour. The astaxanthin levels and red-colour score in the SH6 spore H/M groups were significantly higher than the SH6 spore/L and negative control groups. However, these groups were less efficient than direct supplying astaxanthin or SH6 carotenoids. These data indicate that the preferred dose for this beneficial effect is >1 × 10^6^ CFU/g pellet. Interestingly, we found that SH6 carotenoids at 100-fold lower concentration (5 μg/g pellet) could provide similar increases in these two factors with the synthesized astaxanthin Carophyll (0.5 mg/g pellet). This evidence suggests that natural carotenoids may be better than synthesized ones in terms of absorption and conversion into astaxanthin in shrimp muscle. The positive correlation between shrimp colourisation and astaxanthin concentration in shrimp tissue of the six groups (in descending order, specifically the Carophyll group, SH6 carotenoids, SH6 spore-H, SH6 spore-M, SH6 spore-L and control group), confirmed that astaxanthin plays a key role in shrimp pigmentation and that carotenoids produced by *B*. *aquimaris* SH6 can be successfully absorbed and converted to astaxanthin in white-leg shrimp in a dose-dependent manner. Nevertheless, both Carophyll and SH6 carotenoids have high production costs and are less efficient in terms of weight gain. Thus the carotenoid-producing *B*. *aquimaris* SH6 orange spores seem to be a good choice because production is much more convenient and at a lower cost. Moreover, the colour spores can provide multiple beneficial effects to shrimp, not only astaxanthin and colour improvement, but also on other probiotic activities.

The third probiotic activity of SH6 spores we would like to discuss is immune-stimulatory effects. In this study, a feeding regime at the three different doses commonly used for shrimp in comparison to positive and negative controls was used to show for the first time that the stimulatory effects of *B*. *aquimaris* SH6 spores in white-leg shrimp are significant at the threshold dose of 1 × 10^6^ CFU/g. This was indicated by the increased expression of two molecular markers, *Rho* mRNA expression and PO activity, which are associated with the innate immune status of white-leg shrimp. *Rho* has been reported to be involved in signal transduction in the defence response and is considered a molecular marker for virus-resistant shrimp, and is therefore an indicator of the ability of shrimp cells to defend against pathogen invasion. Expression of the *Rho* gene has been previously found at higher levels in the WSSV-resistant shrimp compared to normal shrimp [30]. In a similar way, our data exhibited the beneficial effects of SH6 spores through the activation of *Rho* mRNA expression to improve shrimp immune system. Here, *Rho* mRNA expression was not completely proportional to the doses fed to shrimp, but the expression level increased significantly 8–9 folds (*P* <0.05) after 28 d in SH6 spore-H/M group, compared to the negative control, while the SH6 spores at low doses had a smaller effect (3-fold, *P* >0.05) (Fig 4A). In addition to *Rho* mRNA expression, we also found that PO activity was dominant in the SH6 spore-H group, followed by the SH6 spore-M group (Fig 2B). In our previous report on the effect of *B*. *aquimaris* SH6 spores and recombinant *Bacillus subtilis* spores expressing the VP28 antigen of white spot syndrome virus (PY79-CotB-VP28) [20,31], we showed that PO activity increased from day 0 to day 7, but remained almost unchanged at day 14. However, these studies have not yet mentioned this index at day 28. Here, the increased PO activity at day 28 was proportional to the dose of SH6 spores, and was typically the highest in the SH6 spore-H group, followed by the SH6 spore-M and SH6 spore-L groups, in comparison to the negative control. These results support the ability of SH6 spores to stimulate the conversional activity of granular cells in pro-PO to PO in white-leg shrimp, which is strengthened over time. This result is similar to a previous study by Pham *et al*. (2014) reporting an improvement in the PO activity of *B*. *subtilis* PY79 and PY79-CotB-VP28 spores at a dose of 1 × 10^6^ CFU/g in the shrimp species *P*. *monodon* [31]. Apart from evaluating *Rho* mRNA expression and PO activity, we also measured another two molecular markers: *Ran* mRNA expression and SOD activity. However, we did not observe any differences in the values between the SH6 spore-feeding groups and the negative control group in terms of these factors. Regarding *Ran* mRNA expression, we unexpectedly detected over-expression in the groups fed directly with Carophyll and SH6 carotenoids, but not in the group fed with SH6 spores. Although this might be due to the carotenoids themselves playing an important role in regulating *Ran* expression, this requires further study. Regarding SOD activity, the level of activity found in the shrimp fed with *Bacillus* spores is a controversial issue and is less consistent between different studies than PO activity. According to Nguyen *et al*., SOD activity has been found to be increased by about 2-fold in white-leg shrimp fed with *B*. *subtilis* PY79 and *B*. *subtilis* PY79-CotB-VP28 spores at extremely high concentrations of 1 × 10^9^ CFU/g pellet [31]. However, in a study by Pham *et al*., SOD activity did not change during the 28-day feeding period in black tiger shrimp fed with the same PY79-CotB-VP28 spores at doses ranging from 10^6^−10^9^ CFU/g pellet [32]. We suspected that SH6 spores affect to the phagocytosis in shrimp not only through the activation of the PO pathway, but also through that of the SOD pathway. Thus, further experiments need to be performed to prove this hypothesis in the future.

Spores of several *B*. *subtilis* strains have been proven to adhere or reside in the gut and subsequently germinate to form biofilm in the small intestine of mice and chickens during dozen-hour transition through the intestine [27,33]. The mechanism by which *Bacillus* spores exhibit their beneficial effects in shrimp is of great interest to scientists, producers and users in the field of shrimp aquaculture because, in general, it is thought that only few-hour transition of food through the shrimp intestine is too quick for feeding with supplements to result in spores to germinating into vegetative bacteria in the shrimp gut, and thereafter secrete carotenoids and useful enzymes. However, until now, there was no available report regarding the colonization and germination of pigmented *Bacillus* strains in the shrimp gut. To our knowledge, this study is the first to demonstrate the colonization and germination of colour *B*. *aquimaris* spores in the gut of white-leg shrimp, resulting in an improvement of total bacterial live-counts and increased diversity in the shrimp gut. To prove that the SH6 spores can reside in the shrimp gut, we hypothesised that if the spores simply transited through the shrimp gut, we would expect the spore CFU to remain relatively constant, since the transit time through the GI-tract is no more than a few hours. However, the evident increase in SH6 CFU in the shrimp gut over time, from day 0 to days 3, 7 and 28, can be considered as evidence that SH6 spores may be able to accumulate in the GI-tract of shrimp, highlighting the successful colonization to the intestinal epithelium. In addition, our data revealed that the total live-counts and diversity of bacterial microbiota in the shrimp gut also improved with increased SH6 counts in the gut during feeding, and in a dose-dependent manner (Fig 1). Interestingly, the major bacteria population we identified all belong to useful bacteria for shrimp aquaculture, which have been either commonly used as probiotic bacteria or are naturally found in the intestines of other aquatic species [5,34–36]. These bacteria include *A*. *venetianus*, *M*. *chocolatum*, *P*. *stutzeri*, *B*. *licheniformis*, *B*. *amyloliquefaciens*, *S*. *amazonensis*, and *S*. *warneri*. Since *B*. *aquimaris* SH6 is the probiotic strain originating from the GITs of white-leg shrimp, it is understandable that this strain has the best chance of surviving and colonizing the intestine, allowing for beneficial microbiota to thrive. The relationship between the dose-dependent probiotic activities with the dose-dependent increased of live-counts and diversity of the gut bacteria suggest that the gut bacteria induced by SH6 spores in the gut play a key role in the beneficial effects to shrimp health.

To confirm that SH6 spores are able to not only colonize the shrimp gut but also thereafter germinate into vegetative cells, we determined the time-dependent expression of the *BaqA*-SH6 gene coding for the α-amylase gene of *B*. *aquimaris* SH6, a molecular marker reflecting spore germination in the gut, for the first time. In general, in a nutrient-rich environment, the germination process of *Bacillus* spores can be activated after a few hours [21,37] and can be detected by measuring the mRNA expression of the gene coding for α-amylase using RT-PCR. For example, according to Casula *et al*., the germination of the recombinant *B*. *subtilis* spore SC2288 starts at 18 h and reaches its highest level at 24 h, at 18%, in the mouse gut [38]. In another study by Stephen *et al*., the germination of spores of the *B*. *subtilis* SC2362 strain started at 12 h and reached saturation at 20 h in both the ceca and colon of the chicken gastrointestinal tract [27]. In our study, we developed a qualitative real-time RT-qPCR for detecting the germination of *B*. *aquimaris* SH6 spores in the shrimp gut using specific primers we designed for the amplification of a 110 bp sequence of the *BaqA*-SH6 gene (Fig 1). Our data revealed that SH6 spores started germinating into vegetative cells at 4 h but that their efficiency remained very low, around 0.04–0.07%, until 12 h. The germination only reached its highest level after 24 h (27.87%) and then reduced gradually from day 2, as we did not supply more feed to the shrimp (Fig 6C). The earlier starting time-point of germination in our study compared to those reported by other research groups in mouse and chicken studies may be due to the fact that the real-time RT-qPCR technique used is able to detect the germination efficiency with high sensitivity, even at 0.04–0.07% efficiencies. With this 4 h-germination starting time, we hypothesised that only the spores colonized in the intestinal epithelium can germinate as they remain in the gut and are thus able to sense the nutrition levels, while free-transit spores are passed through the shrimp gut in under 4 h. Correlating the evidence of dose-dependent increased live counts of SH6 spores with an improved total live-count and diversity of useful bacteria in the shrimp gut, increased immune-associated *Rho* mRNA expression and PO activities, as well as quick and high efficient germination of the spores into vegetative cells in shrimp gut, we strongly believe that the colonization, interaction with shrimp microbiota, and germination of SH6 spores in shrimp gut is the mechanism for beneficial effects of SH6 spores to the host. In theory, the germination of SH6 spores in shrimp is critically important for the host because the vegetative cells can secrete extracellular enzyme such as protease, amylase or lipase to synthesize some growth factors like vitamins, carotenoids, retinols, fatty acids.

## Conclusions

This work is the first demonstration that despite the fact that the transition time of feed through the intestine in shrimp occurs within a few hours, orange-pigmented *B*. *aquimaris* SH6 spores can colonize, accumulate and germinate into vegetative cells in the GI tract, improving the live-counts and diversity of shrimp microbiota as a result. This leads to an innate immune response via increased *Rho* mRNA expression and PO activity and also improves astaxanthin levels, the red-colour score and the growth rate of white-leg shrimp. The probiotic effects of SH6 spores in white-leg shrimp were carefully analysed in a dose-dependent manner, determining that the recommended dose for the optimal probiotic effect of SH6 spores is >1 × 10^6^ CFU/g pellet, and should otherwise not be lower than 2 × 10^5^ CFU/g pellet. In conclusion, our work sheds light on part of mechanism for the beneficial effects of pigmented *Bacillus* spores in shrimp and provides new information about the most cost-effective dose of the pigmented *Bacillus* spore-form probiotics for use as novel feed supplements in shrimp aquaculture.

## Supporting information

S1 Fig *Ran* mRNA expression of *L*. *vannamei* after 28 d feeding with SH6 spores. Experiment includes negative control, Carophyll (astaxanthin: 0.5 mg/g pellet), SH6 carotenoid (SH6 carotenoids: 5 μg/g pellet), and SH6 spore-H/M/L groups (SH6 spores at 5 × 10^6^, 1 × 10^6^, 2 × 10^5^ CFU/g pellet, respectively). Gene expression level of *Ran* gene is indicated by 2^-ΔΔCt^ value.
(TIF)
