# Supplementary figures and images for: Fate of carotenoid-producing Bacillus aquimaris SH6 colour spores in shrimp gut and their dose-dependent probiotic activities
Source: PLoS One. 2018 Dec 21;13(12):e0209341. doi: 10.1371/journal.pone.0209341 (PMC6303041; doi:10.1371/journal.pone.0209341)

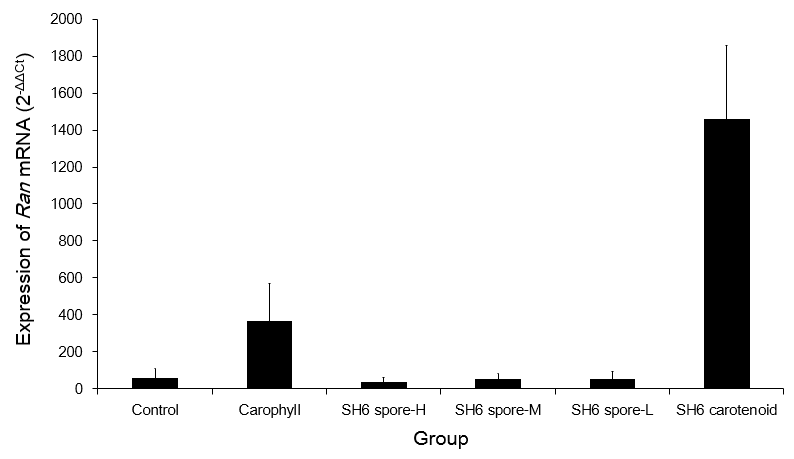

Supplement: S1 Fig — Experiment includes negative control, Carophyll (astaxanthin: 0.5 mg/g pellet), SH6 carotenoid (SH6 carotenoids: 5 μg/g pellet), and SH6 spore-H/M/L groups (SH6 spores at 5 × 10^6^, 1 × 10^6^, 2 × 10^5^ CFU/g pellet, respectively). Gene expression level of Ran gene is indicated by 2-ΔΔCt value. (TIF) [file pone.0209341.s001.tif]
